# An 8-week injury prevention exercise program combined with change-of-direction technique training limits movement patterns associated with anterior cruciate ligament injury risk

**DOI:** 10.1038/s41598-024-53640-w

**Published:** 2024-02-07

**Authors:** M. Mohr, P. Federolf, D. Heinrich, M. Nitschke, C. Raschner, J. Scharbert, A. D. Koelewijn

**Affiliations:** 1 Department of Sport Science, Universität Innsbruck, Fürstenweg 185, 6020 Innsbruck, Austria; 2 Department of Artificial Intelligence in Biomedical Engineering, Friedrich-Alexander-Universität Erlangen-Nürnberg, Erlangen, Germany

**Keywords:** Motor control, Biomedical engineering, Ligaments

## Abstract

Knee ligament sprains are common during change-of-direction (COD) maneuvers in multidirectional team sports. This study aimed to compare the effects of an 8-week injury prevention exercise program containing COD-specific exercises and a similar program containing linear sprint exercises on injury- and performance-related variables during a 135° COD task. We hypothesized that the COD-specific training would lead to (H1) stronger reductions in biomechanical variables associated with anterior cruciate ligament (ACL) injury risk during COD, i.e. knee abduction moment and angle, hip internal rotation angle and lateral trunk lean, and (H2) more effective improvements in COD performance according to the COD completion time, executed angle, ground contact time, and approach speed. Twenty-two sports science students (40% female) completed biomechanical assessments of COD movement strategies before and after participating in two supervised 25-min training sessions per week over 8 weeks. We observed significant ‘training x group’ interaction effects in support of H1: the COD-specific training but not the linear sprint training led to reduced peak knee abduction moments (interaction, *p* = 0.027), initial knee abduction (interaction, *p* < 0.001), and initial lateral trunk lean angles (interaction, *p* < 0.001) compared to baseline. Although the COD-specific training resulted in sharper executed angles (interaction, *p* < 0.001), the sprint-specific training group showed reduced COD completion (interaction, *p* = 0.037) and ground contact times (interaction, *p* < 0.001). In conclusion, a combination of generic and COD-specific injury prevention training resulted in COD technique adaptations that can help to avoid ACL injury-prone COD movements but may negatively affect COD speed.

## Introduction

Acute knee sprains are among the most common sports injuries^1,2^. Ruptures of the anterior cruciate ligament (ACL) are particularly common during evasion sports that require fast change-of-direction (COD) movements, such as sidestepping and pivoting in soccer, basketball, handball, or related sports^3^. Depending on the sport, 50% or more of ACL injuries occur without or with minimal physical contact with the opponent^4,5^. These findings have led to the premise that non-contact ACL injuries often result from poor neuromuscular control and corresponding injury-prone lower extremity and trunk postures—particularly during initial contacts of deceleration and COD maneuvers^5,6^. Injury-prone movement patterns combine lateral trunk lean opposite to the intended direction of travel with joint configurations associated with knee valgus collapse including knee abduction and hip internal rotation during the initial COD stance phase^5,7,8^. The hypothesized mechanism for non-contact ACL injuries during CODs is that the external forces acting on the athlete during such high-intensity turning maneuvers force the femur to slide posteriorly and rotate with respect to the tibia and strain the ACL to the point of failure^9^. Albeit debated, the most important loading components in this mechanism appear to be high axial compressive and shear forces at the knee coupled with knee abduction and internal rotation moments^6,10^. Following this premise, non-contact ACL injuries may be prevented through targeted injury prevention exercise programs (IPEPs) that aim to avoid the hypothesized loading scenario through basic neuromuscular conditioning and technique training^11^. The peak knee abduction moment in particular has been investigated in many previous studies as a surrogate measure of ACL injury risk in athletes^12^ given its association with the ACL loading mechanism^10,13^. In fact, the injury-prone movement patterns mentioned above (lateral trunk lean, knee abduction, hip internal rotation) are all associated with an increased peak knee abduction moment as these movements tend to increase the frontal plane distance between the knee joint and the ground reaction force vector^14^.

A number of IPEPs have demonstrated efficacy in reducing the risk of lower extremity injury risk by up to 50% in sports like soccer and basketball or in physical education^15–17^, with some leading to a reduced risk of ACL ruptures and knee injuries specifically^18–22^. Despite the promising efficacy, it is currently unclear how exactly the completion of IPEPs leads to a reduction of ACL injury risk in participating individuals^23^. For example, there is conflicting evidence whether FIFA11+^15^—one of the best-known IPEPs—actually helps to avoid injury-prone movement strategies during COD maneuvers and thus whether the program is useful to prevent COD-related ACL injuries. While there is some evidence for a reduced knee valgus collapse during a 90° COD maneuver following participation in FIFA11+^24^, the program does not seem to affect the peak knee abduction moment during 45° or 90° COD maneuvers^24–27^. Therefore, the preventative effect of the FIFA11+ with respect to ACL ruptures could be improved if the program included more COD-specific drills^24,28^ as compared to the original version with only one COD-related exercise^29^. In fact, a 6-week isolated COD technique training (30 min sessions twice per week) has already shown potential to improve COD performance and avoid injury-prone movement patterns^30–32^. It is unclear, however, whether similar effects could be achieved with a combined 25-min training program consisting of generic IPEP elements with added COD-specific drills. Such a program may be short enough to be integrated into regular team practices in multidirectional sports^33^ and may result in both the holistic preventative effect of the FIFA11+ program plus more effective prevention of COD-related knee injuries.

Importantly, any IPEP will only be adopted and implemented by coaches and training staff if the program completion does not limit the performance of players^34^. COD performance can be characterized by the ability to perform CODs at high speeds and depending on the situation achieve sharp COD angles to gain spatial advantages over the opponents^35^. COD speed and executed angle are inversely related such that sharper CODs often require slower COD speeds and longer ground contact times given the higher demands on deceleration before and re-acceleration after the COD^36^. With respect to knee injury prevention, some proposed technique modifications such as a narrower foot placement or a ‘softer’ landing with more knee flexion may be beneficial to reduce ACL loading but may be at odds with maximum COD speed and/or sharp COD angles—a phenomenon known as the performance-injury conflict^36,37^. Since previous studies only assessed FIFA11+ effects on submaximal COD maneuvers at angles between 45° and 90°^24–26^, it is unknown whether those findings can be generalized to maximum-speed CODs. In addition, maximum-speed CODs at sharper angles 90°–135° lead to the highest peak knee abduction moments and potentially to the highest risk of knee injury compared to more shallow angles^38^. In summary, there is a lack of research to evaluate the influence of COD-specific IPEPs on performance- and injury-related variables during maximum-speed and sharp COD maneuvers, which may be most relevant to non-contact ACL injury scenarios.

The aim of this study was to investigate the effects of an 8-week IPEP combined with COD-specific exercises (COD training group, CODG) on performance- and injury-related variables during a 135° COD task compared to the effects of an 8-week IPEP combined with linear sprint exercises (sprint training group, SG). The latter condition served as a control group given that linear sprint training is of similar training intensity but is not assumed to improve COD technique^39^. The following hypotheses were tested:

- **H1** The CODG will demonstrate larger pre-IPEP to post-IPEP reductions in biomechanical variables associated with ACL injury risk compared to the SG. These variables included the peak knee abduction moment and the initial knee abduction, hip internal rotation, and lateral trunk lean angles during the COD final foot contact.
- **H2** The CODG will demonstrate larger pre-IPEP to post-IPEP improvements in COD performance compared to the SG. COD performance-related variables included the completion time, the executed angle, the ground contact time, and the approach speed.

## Methods

### Study design and participants

We designed an experimental study to compare the effects of two 8-week neuromuscular training interventions on COD performance and knee joint loading: A basic IPEP plus COD-specific drills (CODG) and a basic IPEP plus linear sprint training (SG). The protocol of this intervention study was pre-registered on ClinicalTrials.gov (ID NCT05014009, first registered on 20/08/2021). We conducted an a-priori sample size estimation based on a previous non-randomized controlled trial reporting a strong, beneficial effect of a 6-week COD technique modification on the Cutting Movement Assessment Score (CMAS), a qualitative screening tool to quantify biomechanical and neuromuscular deficits during COD tasks [significant ‘training x group’ interaction, $\eta_{p}^{2}$ = 0.287]^31^. With an a-priori significance level alpha of 0.05 and a desired power of 0.8, the minimum required sample size was estimated in G*Power v3.1.9.7^40^ as only N = 8 (n = 4 in each training group). The CMAS, however, combines multiple biomechanical and neuromuscular deficits (e.g., knee valgus, lateral trunk flexion, hip internal rotation) into one aggregate score and may thus show a larger effect compared to the analysis of a single biomechanical variable such as the peak knee abduction moment in the current study. Based on these considerations, we selected a desired sample size of N = 24 sports science students (n = 12 per group). With an expected drop-out rate of 10–20%, our sample of at least N = 20 participants was able to detect moderate to strong interaction effects ($\eta_{p}^{2}$ > 0.1) with a power of at least 0.8. The inclusion criteria were (1) sports science student at the University of Innsbruck, (2) 18–40 years old, (3) experience with multi-directional sports that involve CODs (including but not limited to soccer, basketball, volleyball, European handball), and (4) no lower extremity injury in the last six months that led to a disruption of sport participation for at least 2 weeks. Following recruitment, the participants were quasi-randomly allocated to either the CODG or SG. Specifically, weekly training time slots were set to Monday/Wednesday/Friday and Tuesday/Thursday/Friday for the SG and CODG, respectively. Then, participants were assigned to either group based on their availability and weekly schedule, i.e. quasi-randomly, while ensuring a similar representation of males and females per group. This study was approved by the local ethics committee at the Department of Sport Science at the Universität Innsbruck (Board for Ethical Issues, Review Board Sport Science, ID 61/2021) following guidelines on ethical principles for research involving human subjects as outlines in the Declaration of Helsinki. All participants provided written informed consent before commencing the training or measurements.

### Training intervention

Participants were asked to take part in at least two training sessions per week over a duration of 8 weeks. Communication of training schedules and tracking of attendance was achieved within each group using the mobile application SpielerPlus™. All training sessions were supervised by one of two individuals (co-authors MM and JS) with at least an undergraduate degree in sports science plus practical experience in coaching. MM supervised training sessions of the CODG and JS supervised training sessions of the SG. All training sessions had a duration of 25–30 min and took place in an indoor gym. A full description of all training components can be found in Supplementary Table 1.

The first generic training component (10–15 min) was the same for both groups and consisted of eleven exercises of the FIFA11 + IPEP^29^. These exercises include a running and jumping warm-up, strengthening exercises for the core and lower extremity and balance training. Some FIFA11+ exercises such as sidesteps or sprinting were removed from the basic training component because of their overlap with either the COD-specific or sprint-specific training.

Following the generic component, the CODG completed COD-specific exercises and drills (10–15 min) aimed at reducing the risk of knee injury during COD but also improving COD performance. The COD-specific training was largely based on a previously successful 6-week COD technique modification training of Dos’Santos and colleagues^31^ and following other recommendations for training COD, agility, and deceleration^41–43^. Each training session included 1) deceleration exercises, 2) lateral hops and/or drop jumps followed by CODs, and 3) COD drills such as the L-Drill or X-Drill. The difficulty and complexity of the COD exercises increased every 2 weeks based on a gradual introduction of higher speeds, sharper CODs, and elements of uncertainty and competition. During all drills, participants received technique feedback from the supervisor based on movement interventions previously shown to improve performance and/or reduce the peak knee joint moment during COD. Examples include to “slam on the brakes before the COD”, “steer with your upper body” or “land quietly”, aiming to implement an external focus of attention where possible, which has been shown to improve motor learning outcomes^44^.

The SG completed exercises to improve linear sprinting performance, specifically sprint acceleration (10–15 min). The sprint-specific training was based on a previously successful 6-week sprint training program of Lockie and colleagues^45^ and following other recommendations for speed and agility training^41^. Each session included (1) plyometric exercises including variations of drop jumps, skips, and bounds, and (2) variations of sprint starts and early acceleration up to 15 m. Similar to the CODG, the exercises increased in difficulty and complexity every 2 weeks by introducing a larger volume of sprints, longer sprints, and more coordinatively challenging sprinting drills. The SG also received technique feedback aimed at improving sprinting performance such as encouraging short ground contacts.

### Experimental procedures

**Figure 1:**
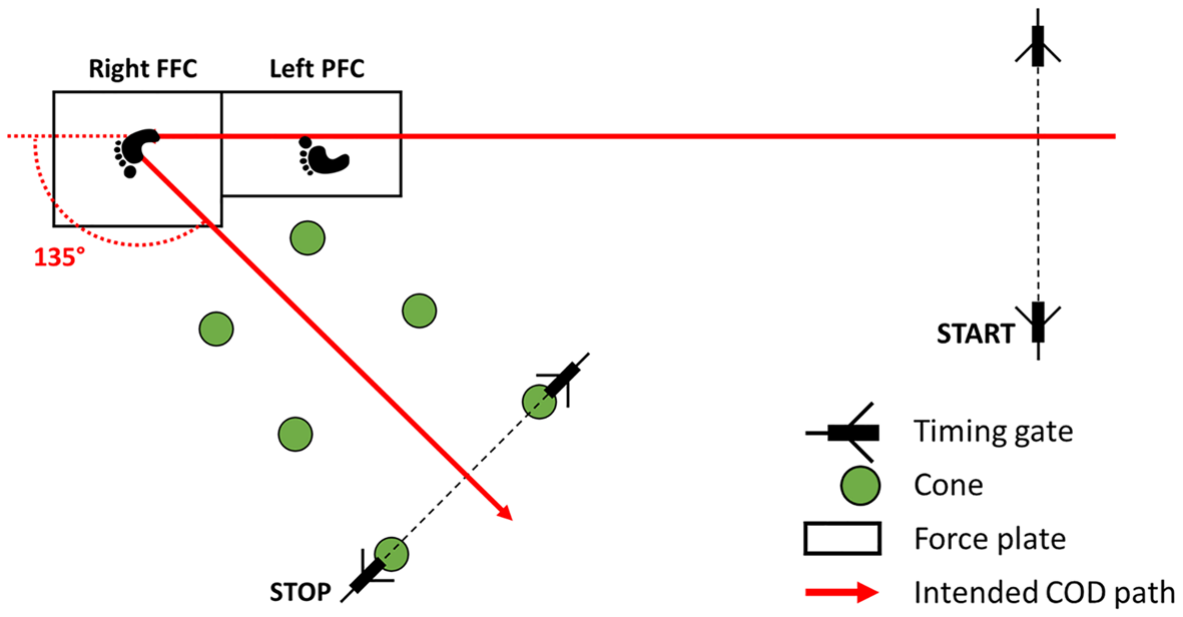
135° COD experimental set-up. *PFC* penultimate foot contact, *FFC* final foot contact.

Baseline and follow-up measurements followed the same procedures and were carried out at the movement laboratory at the Department of Sport Science at the Universität Innsbruck within 1–2 weeks before and after the beginning and end of the training, respectively. During the baseline measurement participants filled out a questionnaire on demographics, basic anthropometrics, sports participation, and previous musculoskeletal injuries. Then, participants were equipped with 56 retro-reflective markers according to a modified version of the Vicon Plug-in Gait fullbody plug-in-gait marker set (see Supplementary Figure 1). Modifications included (1) replacement of the thigh and shank markers with marker clusters including four markers each, (2) addition of medial knee and ankle markers, which were removed after the initial, static standing calibration, (3) two instead of one forefoot markers placed on the participants’ shoes according to the 1st and 5th metatarsal joints, (4) two additional markers on the left and right iliac crest, and (5) removal of the finger markers. Following marker placement, individuals completed a 5-min standardized warm-up on a stationary bicycle. Then, individuals carried out a series of submaximal 45° and 135° CODs for a more specific warm-up and familiarization with the COD task. Following the submaximal warm-up, each participant had to demonstrate at least three successful 135° CODs at maximum speed before the actual testing started. Additional familiarization trials were allowed if required by participants. The criteria for a successful 135° COD were to (1) complete the penultimate (left) foot contact on the first force plate, (2) complete the final (right) foot contact on the second force plate, (3) change direction on the second force plate by 135°, avoiding a “rounded” turn, (4) perform the COD task as fast as possible from the initial to the final timing gate (see Fig. 1). Both force plates were covered with a grid of athletic tape to avoid the risk of slipping.

For the actual data collection, participants stood on the force plate for 2 s in an upright, static posture (‘T-Pose’). Next, participants completed six successful 135° COD movements at their maximum speed with at least 1 min of rest in between recordings. COD completion time was captured by timing gates placed at a distance of 5 m (start) and 2.7 m (stop) from the center of the second force plate (see Fig. 1). The three-dimensional marker trajectories were captured by a 10-camera optical motion capture system (250 Hz sampling rate, VICON, Oxford, UK). Ground reaction forces of the penultimate and final foot contacts were captured by separate force plates (1000 Hz sampling rate, first plate: Kistler, Winterthur, CH; second plate: AMTI, Watertown, MA, USA).

### Data processing

Marker trajectories were reconstructed through VICON Nexus software v2.12 (VICON, Oxford, UK). Then, standard model scaling, inverse kinematics, and inverse dynamics procedures were completed in OpenSim 4.2^46^ using the generic whole-body musculoskeletal model of Catelli and colleagues^47^, which has been optimized for motor tasks involving high knee and hip flexion range of motion. This model originally contains a single degree of freedom knee joint for knee flexion‑extension and was updated to include two additional degrees of freedom for knee abduction‑adduction and knee internal‑external rotation. The ankle pronation‑supination degree of freedom was locked as has been done in previous COD biomechanical analyses^48,49^. The pelvis rotation sequence was updated from pelvis tilt (z), list (x), rotation (y) to pelvis rotation (y), obliquity (x), tilt (z) to allow a clinical interpretation of pelvis rotation independent of the movement direction^50^. A final modification was to unclamp shoulder angles from the range of motion limits given that some participants showed an extreme arm swing motion during the CODs leading to unrealistic jumps in the inverse kinematics solution for shoulder and torso rotations. All further processing steps were done in a custom-written MATLAB (The MathWorks, Inc., Natick, MA, USA) script and the OpenSim Application Programming Interface (API).

The biomechanical analysis for this manuscript focused on the penultimate (PFC) and final foot contacts (FFC), which were detected according to the raw vertical ground reaction forces and a threshold of 20 N^51^. Marker trajectories and force data were filtered using a third-order dual-pass low-pass Butterworth filter with a cut-off frequency at 15 Hz. The cut-off frequency was selected based on a previous study, which determined appropriate cut-off frequencies for marker trajectories during side-cutting tasks^52^ and the recommendation to select equal cut-off frequencies for marker and force data when computing intersegmental moments^51,53^. The following biomechanical variables were further processed: the peak knee abduction moment within the first 25% of FFC and the knee abduction angle, hip internal rotation angle, and lateral trunk lean (i.e. pelvis-trunk angle in the frontal plane) at the initial time of FFC (see Supplementary Figure 2 for average waveforms). Please note that the peak knee abduction moment is an externally applied moment, i.e. the knee joint moment in the frontal plane caused by all external forces (the ground reaction force, inertial forces, etc.). The peak knee abduction moment was normalized to body mass to enable between-subject comparisons. The following performance variables were further processed: The ground contact time of the FFC, the executed COD angle, and the approach speed, i.e. the magnitude of the horizontal center-of-mass (COM) velocity at the initial time of the PFC. The executed COD angle was estimated as the angle between the COM horizontal velocity vector at the beginning of the PFC and the respective vector at the end of the FFC^54^.

### Data analysis and statistics

We used linear mixed effects models clustered by participants to investigate the effects of the factors ‘training’ (baseline vs. follow-up) and ‘group’ (CODG vs. SG) and their interaction (‘training x group’) on our biomechanical and performance outcome variables. Such models allow to fully capture the dispersion of experimental data by including multiple repetitions of COD movements per individual (six COD trials in this case) and thus avoid the issue of having to average over repetitions or select specific repetitions for analysis^55,56^. Furthermore, such models allow to adjust for covariates that vary from one repetition to the next, e.g. approach speed or the executed COD angle, and for potential confounding factors that are constant for a given individual such as sex. For all biomechanical outcomes (pKAM, initial knee abduction, internal rotation, and lateral trunk lean angle) linear mixed effects models were defined as follows:

1 $$\hat{y}_{j} = \overline{a} + \left( {a_{j} - \overline{a}} \right) + \overline{b}_{G} \cdot x_{G} + \overline{b}_{T} \cdot x_{T} + \overline{b}_{T*G} \cdot x_{T*G} + \overline{b}_{Sex} \cdot x_{Sex} + \overline{b}_{S} \cdot x_{S} + \overline{b}_{A} \cdot x_{A} + \epsilon_{j}$$

were $\hat{y}_{j}$ is the predicted biomechanical outcome for a given cluster of COD trials from one individual *j,*$\overline{a}$ and $a_{j}$ are the average and individual-dependent intercepts, $\overline{b}$ are the average coefficients across individuals for each predictor variable (G = group, T = training, T*G = training x group interaction, sex, S = approach speed, A = COD executed angle), and $\epsilon$ is an error term representing unexplained variance. All predictor variables were treated as fixed effects, which means their corresponding coefficients were constant across all participants. The model intercept was treated as a random effect, i.e. the intercept varied across participants to take into account the dependence of observations from multiple COD trials within each participant. We included the sex of participants as a factor due to the small difference in the proportion of female participants between training groups (see Table 1) and previously reported sex-specific COD movement patterns^57^.

**Table 1:** Participant characteristics.

Variable	SG n = 11	CODG n = 11
Proportion of female participants (%)	45	36
Age (years)	22.3 ± 1.6	24.3 ± 2.0
Height (cm)	174 ± 12	176 ± 10
Body mass (kg)	66 ± 9	71 ± 8
Frequency of participation in multi-directional sports per week	1.8 ± 1.1	2.0 ± 1.1
Frequency of participation in all sports per week	5.3 ± 1.3	5.2 ± 1.4
Training experience in multi-directional sports in years	10.1 ± 6.7	7.9 ± 5.5
Frequency of IPEP training sessions per week during intervention study	1.7 ± 0.4	1.4 ± 0.3

*All variables except ‘Proportion of female participants’ show the mean ± standard deviation.

Similarly, for all performance outcomes (COD completion time, COD executed angle, ground contact time, and approach speed), linear mixed effects models were defined in accordance with Eq. (1) but without approach speed and COD executed angle as covariates:

2 $$\hat{y}_{j} = \overline{a} + \left( {a_{j} - \overline{a}} \right) + \overline{b}_{G} \cdot x_{G} + \overline{b}_{T} \cdot x_{T} + \overline{b}_{T*G} \cdot x_{T*G} + \overline{b}_{Sex} \cdot x_{Sex} + \epsilon_{j}$$

The model assumptions of normally distributed residuals as well as constant variance of residuals (absence of heteroscedasticity) were investigated and confirmed based on Kolmogorov–Smirnov tests of residuals and scatter plots of predicted vs. residual values^58^. Model outputs included (1) the marginal and conditional R-squared value, which describes the proportion of variance in the outcome variable explained by the fixed and random effects, respectively, (2) F-tests associated with the fixed effects, which can be interpreted analogous to a classic analysis of variance table^59^, (3) fixed effects model coefficients and 95% confidence intervals, and (4) estimated marginal means and 95% confidence intervals for each level of the ‘training’ and ‘group’ factors. In the presence of significant effects of ‘training’ or ‘training x group’ interaction effects, we conducted a simple effects analysis to investigate significant training effects specific to each training group. Cohen’s d effect sizes were determined from the t-statistic of the simple effects analysis following the equation provided by Lakens^60^ and were interpreted according to guidelines from Cohen^61^ (d < 0.2 negligible effect, 0.2 ≤ d < 0.5 small effect, 0.5 ≤ d < 0.8 moderate effect, d ≥ 0.8 large effect).

We rejected the null hypotheses if there were significant ‘training x group’ interactions where the simple effects analyses indicated more favorable training outcomes in the CODG compared to the SG. All statistical analyses were conducted in jamovi software (The jamovi project (2023). *Jamovi* (Version 2.3.21) using the *GAMLj* module (version 2.6.6)^59^ with its default settings (simple factors coding and centered covariates).

## Results

### Participant characteristics and training adherence

Table 1 shows participant characteristics and training adherence. One individual per group dropped out of the study due to illness, leading to n = 11 participants included per group in the statistical analysis. On average, training adherence was higher in the SG (mean ± SD: 1.7 ± 0.4 training sessions per week) compared to the CODG (1.4 ± 0.3 training sessions per week).

### Peak knee abduction moment

**Figure 2:**
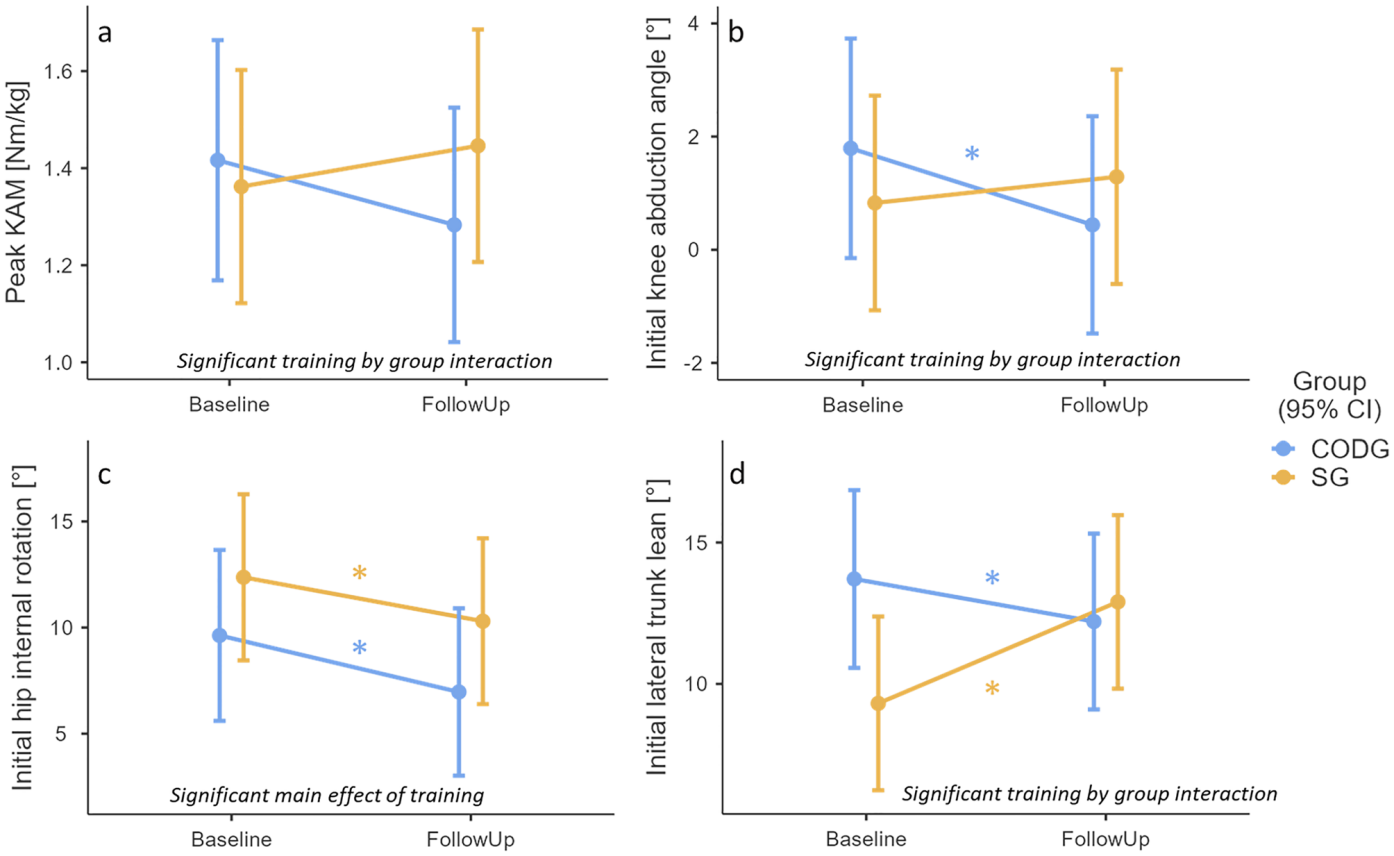
Training effects on COD biomechanical variables associated with ACL injury scenarios during COD. Subpanels show the estimated marginal means and 95% confidence intervals (CI) of the COD-specific IPEP (CODG, blue) and sprint-specific IPEP (SG, orange) for the peak knee abduction moment (pKAM, **a**), initial knee abduction angle (**b**), initial hip internal rotation angle (**c**), and initial lateral trunk lean (**d**) during the baseline and follow-up assessment. Asterisks mark statistically significant changes from baseline to follow-up. *Please note that these means are adjusted for the factor sex and covariates COD approach speed and COD angle.*

There was a significant ‘training x group’ interaction effect (F(1,187) = 4.997, *p* = 0.027) with respect to the peak knee abduction moment (Fig. 2a). The simple effects analysis did not show statistically significant training effects within each specific group but the CODG showed an average reduction with a moderate effect size (mean [95% confidence intervals] of follow-up minus baseline peak knee abduction moment: − 0.13 [− 0.27, 0.01] Nm/kg, *p* = 0.063, d = 0.56). The SG showed an average increase with a small effect size (0.08 [− 0.05, 0.22] Nm/kg, *p* = 0.209, d = 0.38) in the peak knee abduction moment from baseline to follow-up.

### Initial joint angles

The initial knee abduction angle showed a significant ‘training x group’ interaction effect (F(1,185) = 13.449, *p* < 0.001) (Fig. 2b). According to the simple effects analysis, the CODG showed a significant reduction in the initial knee abduction angle at follow-up with a large effect size (mean [95% confidence intervals] of follow-up minus baseline knee abduction angle: − 1.35 [− 2.07, − 0.64] °, *p* < 0.001, d = 1.12). The SG showed a non-significant increase and a small effect size in the initial knee abduction angle from baseline to follow-up (0.46 [− 0.21, 1.13] °, *p* = 0.175, d = 0.41).

With respect to the initial hip internal rotation angle, there was no significant ‘training x group’ interaction effect (F(1,185) = 0.157, *p* = 0.692) but a significant main effect of ‘training’ (F(1,18.6) = 9.704, *p* = 0.002) (Fig. 2c). Simple effects analysis indicated that the hip internal rotation angle was significantly reduced for both groups at follow-up with moderate effect sizes (CODG: mean [95% confidence intervals] of follow-up minus baseline hip internal rotation angle: − 2.67 [− 4.84, − 0.49] °, *p* = 0.017, d = 0.73; SG: − 2.07 [− 4.11, − 0.03] °, *p* = 0.047, d = 0.60).

The initial lateral trunk lean showed a significant ‘training x group’ interaction effect (F(1,185) = 35.668, *p* < 0.001) (Fig. 2d). According to the simple effects analysis, the CODG showed a significant reduction in the initial lateral trunk lean at follow-up with a moderate effect size (mean [95% confidence intervals] of follow-up minus baseline lateral trunk lean: − 1.50 [− 2.74, − 0.27] °, *p* = 0.017, d = 0.72). The SG showed a significant increase with a large effect size in the lateral trunk lean from baseline to follow-up (3.59 [2.44, 4.74] °, *p* < 0.001, d = 1.85).

### COD performance

**Figure 3:**
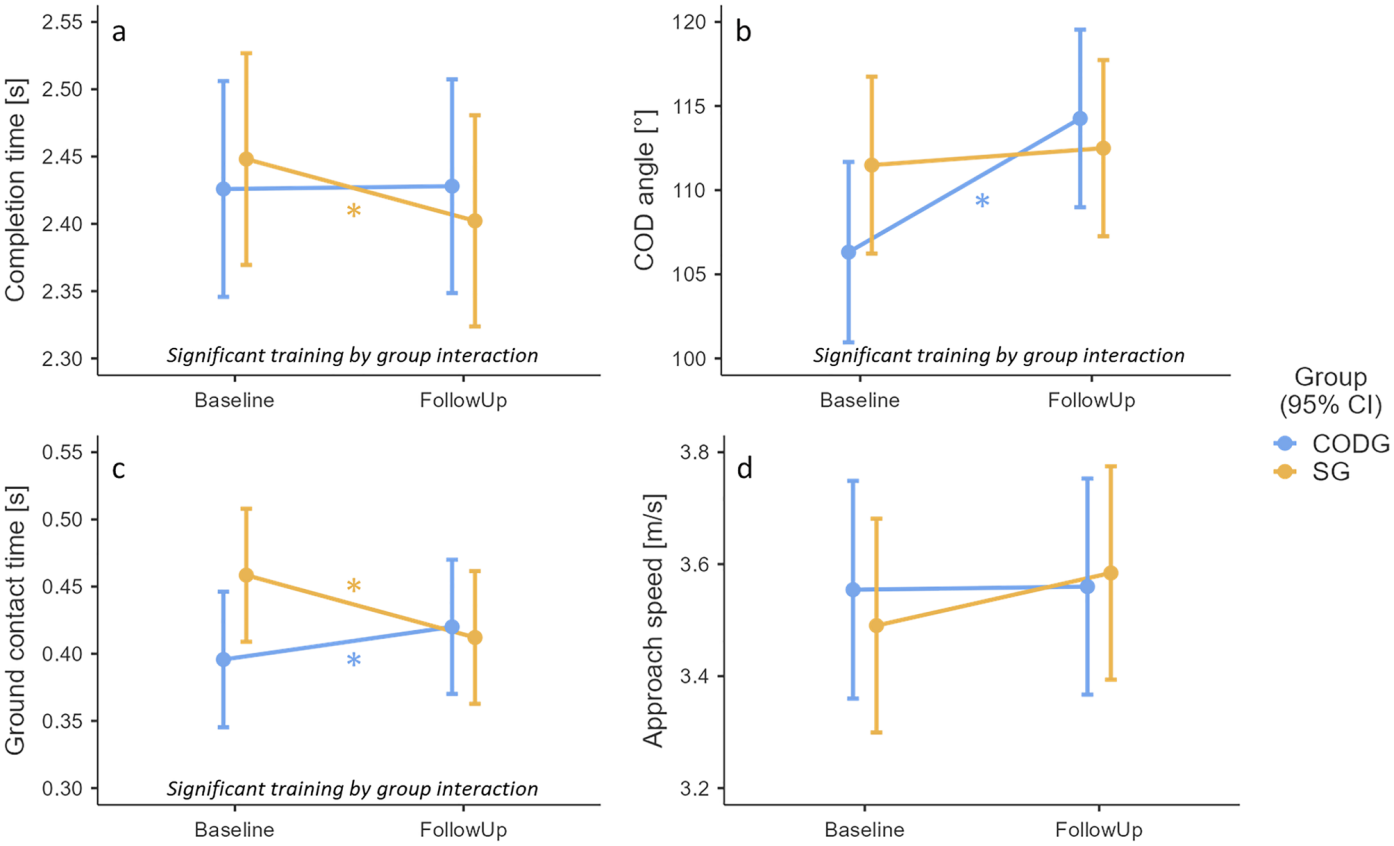
Training effects on COD performance variables. Subpanels show the estimated marginal means and 95% confidence intervals (CI) of the COD-specific IPEP (CODG, blue) and sprint-specific IPEP (SG, orange) for the COD completion time (**a**), COD executed angle (**b**), ground contact time (**c**), and the approach speed (**d**) during the baseline and follow-up assessment. Asterisks mark statistically significant changes from baseline to follow-up.* Please note that these means are adjusted for the factor sex.*

With respect to COD completion times, there was a significant ‘training x group’ interaction effect (F(1,185) = 4.398, *p* = 0.037) (Fig. 3a). The simple effects analysis indicated, that the CODG showed minimal average changes in the completion time from baseline to follow-up with a negligible effect size (mean [95% confidence intervals] of follow-up minus baseline completion time: 0.00 [− 0.03, 0.03] s, *p* = 0.897, d = 0.04). The SG showed a significant reduction in COD completion times at follow-up with a large effect size (− 0.05 [− 0.08, − 0.01] s, *p* = 0.005, d = 0.84).

With respect to COD executed angle, there was a significant ‘training x group’ interaction effect (F(1,185) = 12.69, *p* < 0.001) (Fig. 3b). The simple effects analysis indicated, that the CODG showed a significant increase in the COD angle from baseline to follow-up with a large effect size (mean [95% confidence intervals] of follow-up minus baseline COD angle: 7.94 [5.22, 10.67] °, *p* < 0.001, d = 1.74). The SG showed a non-significant reduction in the COD angle at follow-up with a small effect size (1.01 [− 1.71, 3.72] °, *p* = 0.465, d = 0.22).

The ground contact time showed a significant ‘training x group’ interaction effect (F(1,185) = 21.75, *p* < 0.001) (Fig. 3c). The simple effects analysis indicated, that the CODG showed a significant increase in the ground contact time from baseline to follow-up with a moderate effect size (mean [95% confidence intervals] of follow-up minus baseline contact time: 0.024 [0.003, 0.046] s, *p* < 0.025, d = 0.68). The SG showed a significant reduction in the ground contact time at follow-up with a large effect size (− 0.046 [− 0.067, − 0.025], *p* < 0.001, d = 1.31).

The COD approach speed did not show a significant ‘training x group’ interaction effect (F(1,185) = 2.876, *p* = 0.092) nor a significant effect of ‘training’ (F(1,185) = 3.647, *p* = 0.058) (Fig. 3d). The estimated marginal means in Fig. 3d, however, hint at a trend towards increased approach speeds at follow-up for the SG with minimal average changes in approach speed for the CODG.

### The influence of sex, COD executed angle, and approach speed

A summary of all estimated fixed effects coefficients and corresponding explained variance for each model can be found in the Supplementary Table 2. There was a significant association between sex and two biomechanical variables (pKAM, initial hip internal rotation) as well as one performance variable (COD completion time). The only significant association between biomechanical variables and COD angle/approach speed was found between COD executed angle and the initial knee abduction angle.

## Discussion

The goal of this study was to investigate whether an 8-week IPEP including COD-specific exercises is more effective in limiting joint movements and external loading associated with ACL injury risk during a COD maneuver in comparison to a non-COD-specific IPEP while also improving COD performance. For this purpose, sports science students participated in an 8-week IPEP combined with either COD-specific exercises or linear sprint exercises and their movement patterns during 135° CODs were assessed pre- and post-training.

### Training effects on COD biomechanical variables associated with ACL injury risk

Our first hypothesis that the COD-specific training will demonstrate larger reductions in biomechanical variables associated with ACL injury risk compared to the non-COD-specific IPEP was supported. This is based on the finding that three out of the four investigated variables (peak knee abduction moment, initial knee abduction angle, initial lateral trunk lean) showed a significant ‘training x group’ interaction effect with more favorable training adaptations in the COD-specific training group. The specific adaptations in the COD movement strategy in response to the COD-targeted IPEP were (1) a moderate reduction in lateral trunk lean opposite to the intended movement direction, (2) a moderately reduced knee abduction at initial contact, and (3) a moderately reduced peak knee abduction moment, however, the latter failed to reach statistical significance (*p* = 0.063). Both knee abduction and lateral trunk lean against the cutting direction have been commonly observed in video analyses of non-contact ACL injuries^5,7^ and are known to be associated with the peak knee abduction moment^14^. These findings are in contrast to previously reported effects of generic IPEPs such as the FIFA11+^24,25,27^, which in isolation did not lead to specific changes in the knee abduction angle or moment. However, our findings are in agreement with previous targeted COD technique training reporting reduced lateral trunk lean following training^31,32,43^. For example, Dempsey and colleagues^32^ showed an average reduction in lateral trunk lean from 7.4° to 3.9° following 6 weeks of COD technique training with two 15-min sessions per week. The higher average trunk lean in the current study (10°–15°, see Fig. 2b) is likely due to the sharper COD angle in the current study (135° vs. 45° COD)^62^. The stronger average reduction in the previous compared to the current study (3.5° vs. 1.5°) may show the benefit of providing immediate video feedback to participants as was done in the previous study. This larger reduction in lateral trunk lean can also partially explain why Dempsey and colleagues observed a more conclusive (36%) reduction in the peak knee abduction moment compared to the non-significant (10%) reduction in the current study. Other factors that may have dampened the effect sizes of within-group movement adaptations in our study are (1) the relatively low training adherence within the CODG (1.4 instead of the targeted two training sessions per week) and (2) the high frequency of participation in other sports next to the training intervention (average of five additional physical activity sessions per week, see Table 1), which is likely a unique characteristic of sport science students and could blur the intended training effect. It can be hypothesized that a neuromuscular training frequency of two to three sessions per week and a stricter training schedule would lead to larger effect sizes^63^.

Nevertheless, the results show that as few as eleven training sessions over an 8-week period of a COD-specific IPEP can help to avoid knee and trunk biomechanical patterns during CODs associated with the ACL injury mechanism. This is in contrast to generic IPEPs such as FIFA11+, which do not seem to produce such adaptations. It is important to note, however, that the predictive value of the peak knee abduction moment for ACL injury risk is debated^64^. This is due to the fact that ACL tissue loading depends on many other knee joint loading variables next to frontal plane moments such as the internal rotation moment as well as vertical and horizontal joint reaction forces^9^, which were not considered in this study. While the peak knee abduction moments measured in the current study (80–120 Nm for a body mass of 70kg) were large enough to induce additional ACL strain^65^, it is unknown whether or by how much the observed training-related COD movement adaptations ultimately influenced the ACL strain in our participants.

In contrast, the non-COD-specific training group with a linear sprint focus showed a large increase in lateral trunk lean and a non-significant increase in both the knee abduction angle (moderate effect) and peak knee abduction moment (small effect) and thus a more ACL injury-prone movement pattern. These results are somewhat in agreement with two previous neuromuscular training studies^39,66^, where the respective control groups also showed an increase in COD peak knee abduction moments from pre-to-post training. Notably, the control group in the study of Donnelly and colleagues^39^ also completed linear sprint training similar to the SG of the current study. Furthermore, the training group-independent reduction in the hip internal rotation angle (Fig. 2c) at the follow-up measurement is consistent with previous training intervention studies related to COD movements^24,27^. Therefore, this result could originate from a familiarization effect where individuals in any group learn over time to pre-rotate their pelvis to initiate the COD before the final foot contact and thus reduce energy absorption through the hip joint^67^. The other option is that the basic neuromuscular training components done by both training groups improved hip muscle function and helped to limit hip internal rotation of both groups at follow-up. In conjunction, these findings suggest that linear sprint training may induce COD movement adaptations associated with a higher risk of ACL injury and warrant the recommendation that linear sprint training in multidirectional team sports should be combined or alternated with COD technique training to limit knee joint loads during high-speed CODs. Furthermore, these results underscore that a linear sprint training intervention cannot be treated as a true control condition when evaluating the effects of COD technique modification training given the observed adverse effects of sprint training on COD technique.

### Training effects on COD performance

Contrary to our second hypothesis, the COD-specific IPEP did not generally lead to improved COD performance. The one exception was the COD executed angle, for which the CODG showed a large average improvement of 8° at the follow-up measurement with minimal average changes in the SG. This improvement likely mirrors the training of increasingly sharp cuts in the COD-specific IPEP, which were not practiced in the sprint-specific IPEP. However, both training groups fell short of the 135° COD target post-training, which is consistent with previous analyses across a range of COD angles^31,35^. Opposite to our hypothesis, the sprint-specific IPEP resulted in a reduced COD completion time and ground contact time without such an effect in the COD-specific training group. The average reduction in ground contact time of the SG was of similar magnitude compared to the reduction in completion time (both about 50 ms) and thus the first likely explains the latter^30^ and reflects the practice of quick contacts in the linear sprint group. Furthermore, the SG showed a trend towards faster approach speeds post-training, which may have resulted from the training focus on early acceleration ability in this group. At the same time, the higher approach speed could be one factor to explain the adverse effects on COD technique observed for this group such as higher lateral trunk lean at initial foot contact. Given that sprint training mostly focuses on trunk positioning in the sagittal plane^41^, individuals in the SG may have not been able to properly control frontal plane trunk motion during the COD at higher approach speeds. Opposite to the SG, the CODG showed moderately longer ground contact times and thus reduced performance, which is inconsistent with two prior 6-week COD technique modification interventions^30,31^. The lack of faster times in the CODG may again stem from the low training adherence or parallel physical activity participation but could also reflect the angle-velocity trade-off^36^ where the improved COD executed angle in the CODG resulted in compromised COD speed.

### Limitations and outlook

Here, we demonstrate training effects of a shortened version of the FIFA11+ program combined with either COD technique training or sprint training on COD movement patterns. Our study design was limited in the sense that (1) it could not conclusively distinguish between FIFA11 + effects versus COD/sprint training effects and (2) it cannot reveal whether the short FIFA11+ component retains the efficacy of the original FIFA 11+ to reduce the risk of sports-related injuries across a range of sports^15^. However, given the many ‘training x group’ interactions with respect to our investigated variables, we assume that most of the observed adaptations are the result of completing COD technique training vs. sprint training rather than the basic neuromuscular training components of the shortened FIFA11+ shared across groups. Furthermore, other authors have provided evidence that shortened^68^ or re-scheduled^69^ versions of the original FIFA11+ program retained their protective effect against a range of acute and overuse lower extremity injuries in football athletes. Therefore, we hypothesize that multidirectional team sport athletes who regularly perform a 25–30 min combined training program consisting of generic IPEP elements and COD-specific drills can get ‘the best of both worlds’: (1) a more effective prevention of ACL injuries during COD maneuvers compared to a generic IPEP alone but (2) still retain the benefits of generic IPEPs for preventing acute and overuse sports injuries other than ACL ruptures. This hypothesis should be tested in a follow-up prospective trial to assess the efficacy of a COD-specific IPEP on reducing the risk of sports injury—ACL injuries in particular—in comparison to a generic IPEP alone.

## Conclusion

After completing an 8-week injury prevention exercise program that included both generic neuromuscular exercises and specific exercises to improve COD technique, individuals were able to perform 135° CODs at sharper angles but with lower initial knee abduction, lower lateral trunk lean and a non-significant but moderate reduction in the peak knee abduction moment. Based on previous evidence, these training-related technique modifications can help to avoid injury-prone COD movements but came at the expense of longer ground contact times. A second training group that completed the same generic neuromuscular exercises but specific exercises to improve linear sprinting performance showed improved COD performance (reduced ground contact times) but adverse effects on COD technique, which warrants that sprint and COD technique training should always be combined in multi-directional sports to avoid injury-prone COD maneuvers while maintaining COD performance at increasingly high running speeds.

### Supplementary Information

Supplementary Information 1.

Supplementary Information 2.

Supplementary Information 3.

Supplementary Information 4.

## Data Availability

The dataset generated during and/or analyzed during the current study are available in the Mendeley data repository [10.17632/m4fdzyth8w.2]. The published dataset includes all processed outcome variables and anonymized participant information. The raw data underlying the current study are not publicly available at the time of publication of this article due to confidentiality agreements within an ongoing research project but the raw data are available from the corresponding author on reasonable request.
