# Author Correction: An 8-week injury prevention exercise program combined with change-of-direction technique training limits movement patterns associated with anterior cruciate ligament injury risk

**DOI:** 10.1038/s41598-024-56031-3

**Published:** 2024-03-05

**Authors:** M. Mohr, P. Federolf, D. Heinrich, M. Nitschke, C. Raschner, J. Scharbert, A. D. Koelewijn

**Affiliations:** 1 Department of Sport Science, Universität Innsbruck, Fürstenweg 185, 6020 Innsbruck, Austria; 2 Department of Artificial Intelligence in Biomedical Engineering, Friedrich-Alexander-Universität Erlangen-Nürnberg, Erlangen, Germany

Correction to: *Scientific Reports* 10.1038/s41598-024-53640-w, published online 07 February 2024

The Funding section in the original version of this Article was omitted. The Funding section now reads:

“This work was co-funded by the Austrian Science Fund FWF (M.M., project number I 6547-B) and the German Research Foundation DFG (A.K., project number 520189992).”

The original Article has been corrected.

