# Supplementary material for: An 8-week injury prevention exercise program combined with change-of-direction technique training limits movement patterns associated with anterior cruciate ligament injury risk
Source: Sci Rep. 2024 Feb 7;14:3115. doi: 10.1038/s41598-024-53640-w (PMC10850483; doi:10.1038/s41598-024-53640-w)
Supplement: Supplementary file 1 — Supplementary Information 1. [file 41598_2024_53640_MOESM1_ESM.pdf]

Supplementary File for Article:

An 8-week injury prevention exercise program combined with change-of-direction technique training limits movement patterns associated with anterior cruciate ligament injury risk.

Authors: Mohr M, Federolf P, Heinrich D, Nitschke M, Raschner C, Scharbert J, Koelewijn AD

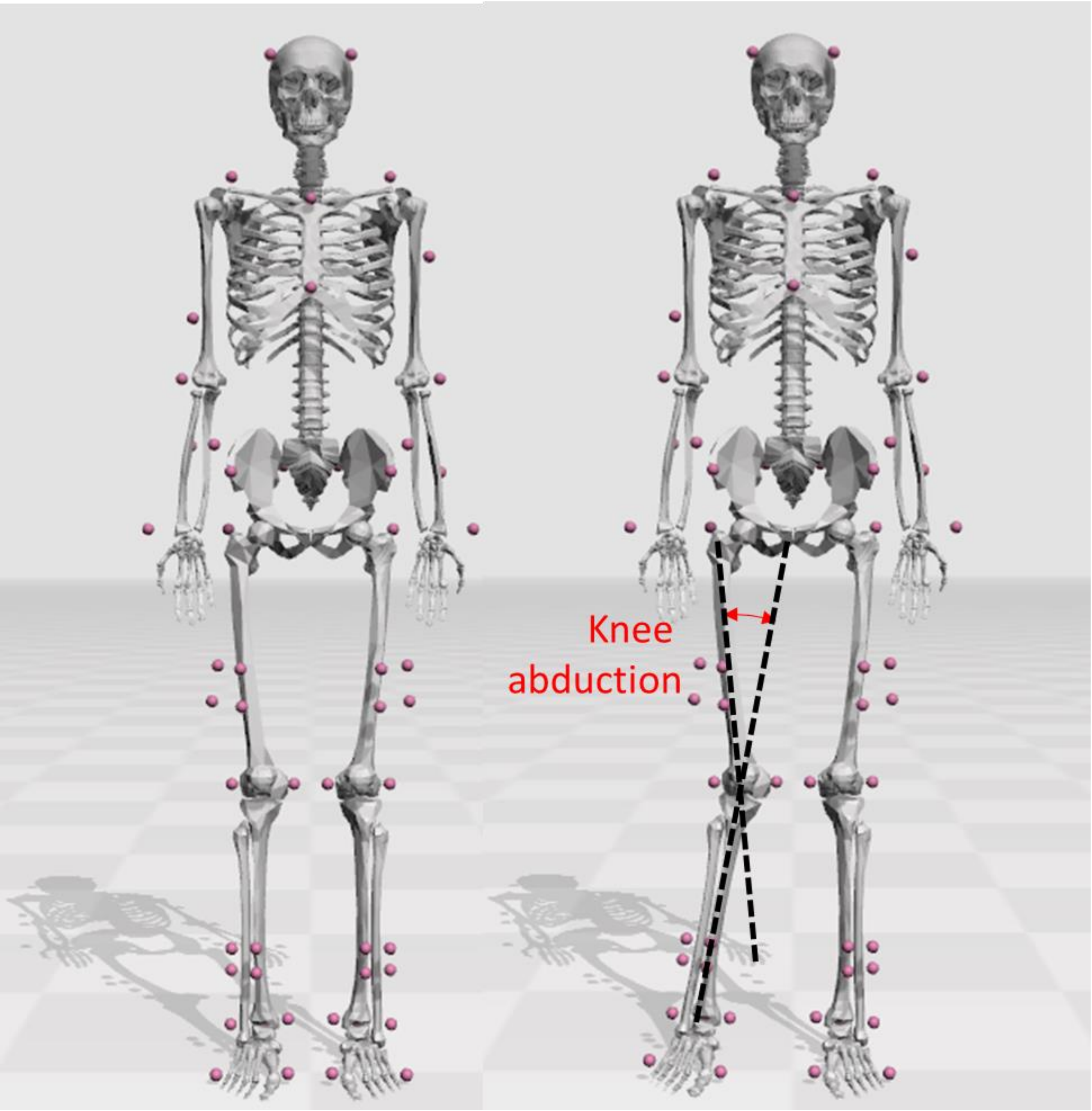

Supplementary Figure 1: OpenSim biomechanical model. The left and right avatars show the Catelli OpenSim model and virtual markers utilized in the current study with a neutral knee abduction/adduction angle (left) and a 10° knee abduction angle for the right leg (right avatar). This figure was created in OpenSim Creator (Kewley, A., van Beesel, J., & Seth, A. (2023). OpenSim Creator (Version 0.5.5) [Computer software]. https://github.com/ComputationalBiomechanicsLab/opensim-creator).
