# Supplementary material for: An 8-week injury prevention exercise program combined with change-of-direction technique training limits movement patterns associated with anterior cruciate ligament injury risk
Source: Sci Rep. 2024 Feb 7;14:3115. doi: 10.1038/s41598-024-53640-w (PMC10850483; doi:10.1038/s41598-024-53640-w)
Supplement: Supplementary file 2 — Supplementary Information 2. [file 41598_2024_53640_MOESM2_ESM.pdf]

Supplementary File for Article:

An 8-week injury prevention exercise program combined with change-of-direction technique training limits movement patterns associated with anterior cruciate ligament injury risk.

Authors: Mohr M, Federolf P, Heinrich D, Nitschke M, Raschner C, Scharbert J, Koelewijn AD

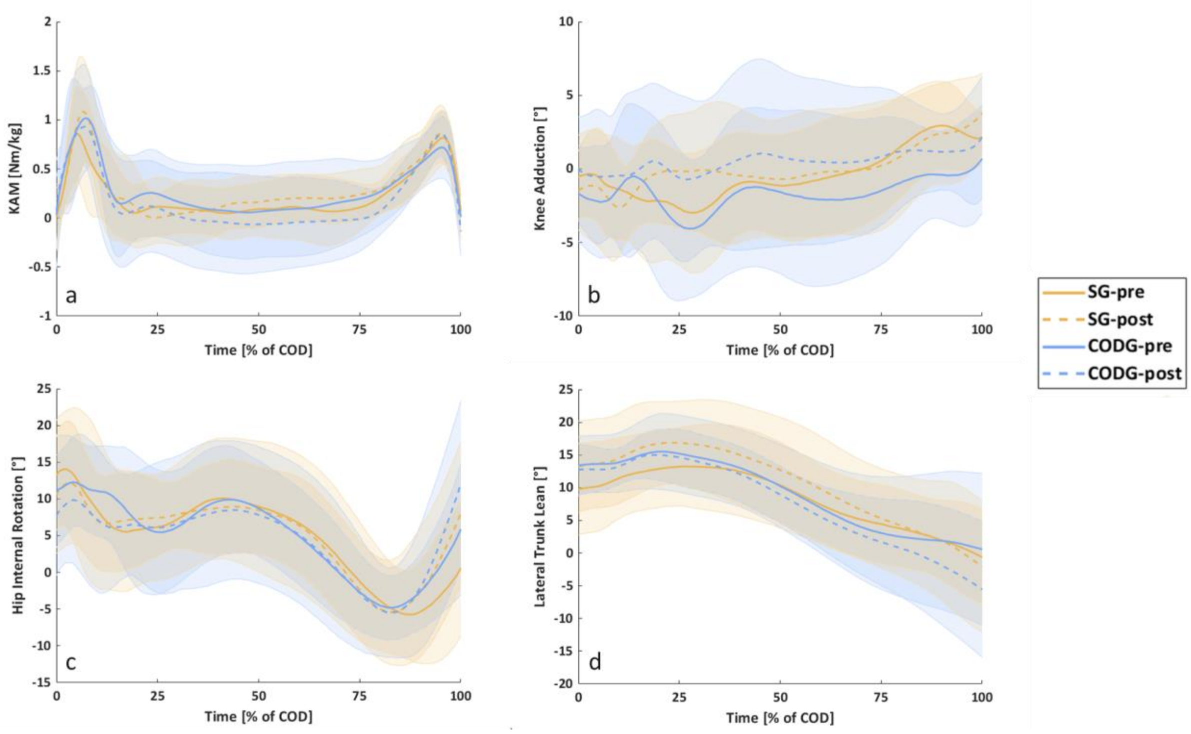

Supplementary Figure 2: Average waveforms of investigated COD biomechanical variables. The subpanels show the mean and standard deviation across participants of the COD-specific (blue) and sprint-specific (orange) training groups pre- (solid) and post-training (dashed) for the knee abduction moment (KAM, a), knee adduction/abduction (b, positive values indicate knee adduction), hip internal/external rotation (c, positive values indicate internal rotation), and lateral trunk lean (d, positive values indicate trunk lean against the intended movement direction). The time axis is normalized to the duration of the COD final foot contact with 0% and 100% representing initial contact and toe-off, respectively. *Please note that the peak KAM values in (a) do not agree with the peak KAM values reported in the main manuscript due to the attenuation effects when averaging waveforms across participants vs. averaging peak KAM values across participants.*
