# Supplementary material for: An 8-week injury prevention exercise program combined with change-of-direction technique training limits movement patterns associated with anterior cruciate ligament injury risk
Source: Sci Rep. 2024 Feb 7;14:3115. doi: 10.1038/s41598-024-53640-w (PMC10850483; doi:10.1038/s41598-024-53640-w)
Supplement: Supplementary file 4 — Supplementary Information 4. [file 41598_2024_53640_MOESM4_ESM.pdf]

Supplementary File for Article:

An 8-week injury prevention exercise program combined with change-of-direction technique training limits movement patterns associated with anterior cruciate ligament injury risk.

Authors: Mohr M, Federolf P, Heinrich D, Nitschke M, Raschner C, Scharbert J, Koelewijn AD

Supplementary Table 2: Fixed effects coefficients and 95% confidence intervals [CI] for each outcome variable and all predictor variables.

	Outcome variable	Marginal R-Squared	Conditional R-Squared	Fixed effects coefficients [95% CI] Group*,§	Time*,§	Group x Time*	Sex*	COD angle	Approach speed
Biomechanical variables	Peak knee abduction moment [Nm/kg]	0.18	0.60	0.05 [-0.25, 0.36]	-0.02 [-0.12, 0.07]	**0.22 [0.03, 0.41]**	**0.37 [0.06, 0.70]**	0.00 [-0.01, 0.01]	0.04 [-0.20, 0.28]
	Initial knee abduction angle [°]	0.13	0.78	-0.06 [-2.54, 2.42]	-0.45 [-0.94, 0.04]	**1.82 [0.85, 2.79]**	-2.19 [-4.73, 0.36]	**-0.04 [-0.07, -0.00]**	0.57 [-0.70, 1.85]
	Initial hip rotation angle [°]	0.28	0.68	3.04 [-2.00, 8.01]	**-2.37 [-3.86, -0.89]**	0.60 [-2.35, 3.55]	**8.59 [3.39, 13.79]**	0.01 [-0.10, 0.11]	1.66 [-2.05, 5.38]
	Initial trunk lean angle [Nm/kg]	0.20	0.78	-1.86 [-5.87, 2.16]	**1.04 [0.20, 1.89]**	**5.10 [3.42, 6.77]**	4.32 [0.20, 8.43]	-0.02 [-0.08, 0.04]	0.49 [-1.70, 2.67]
Performance variables	COD completion time [s]	0.29	0.77	-0.00 [-0.10, 0.10]	-0.02 [-0.04, 0.00]	**-0.05 [-0.09, -0.00]**	**-0.18 [-0.29, -0.08]**	*not applicable* $	
	COD executed angle [°]	0.09	0.59	1.70 [-5.04, 8.45]	**4.48 [2.57, 6.38]**	**-6.94 [-10.76, -3.12]**	2.77 [-4.08, 9.63]		
	Ground contact time [s]	0.06	0.68	0.027 [-0.037, 0.092]	-0,011 [-0.026, 0.004]	**-0.071 [-0.100, -0.041]**	0.003 [-0.062, 0.069]		
	Approach speed [m/s]	0.13	0.75	-0.02 [-0.27, 0.23]	0.05 [-0.00, 0.10]	0.09 [-0.01, 0.20]	0.26 [0.01, 0.51]		

* Variable definitions: Group: SG – CODG, Time: Follow-up – Baseline, Group x Time: SG – CODG x Follow-up – Baseline, Sex: Male – Female

§ In the presence of a significant 'Group x Time' interaction effect, the grey-colored Group and Time effects were not considered

$ COD angle and approach speed were not included as covariates for the analysis of performance variables.

Note: Values formatted in bold indicate a significant association ($p < 0.05$) between the predictor and outcome variable.
